# *Acorus calamus* L. for Cognitive Enhancement: A Systematic Review of Its Ethnobotanical Practices and Scientific Evidence

**DOI:** 10.1002/cbdv.71859

**Published:** 2026-10-26

**Authors:** Raghu Tamang, Anindita Gogoi, Twahira Begum, Mohan Lal

**Affiliations:** ^1^ Agrotechnology and Rural Development Division CSIR‐North East Institute of Science and Technology (NEIST) Jorhat India; ^2^ Academy of Scientific and Innovative Research (AcSIR) Ghaziabad India

**Keywords:** *Acorus calamus*, cognitive enhancement, ethnobotany, pharmacology, phytochemistry

## Abstract

Introduction: *Acorus calamus* L. is an important medicinal aromatic plant belonging to Acoraceae family. The current review article underscores the ethnobotanical significance along with scientific evidence on cognitive and neurological properties of *A. calamus*. Besides these, the article reports the morphology, phytochemistry and biological activities of *A. calamus*. Methods: Using PRISMA (2020) guidelines, an extensive literature review have been searched using PubChem, Google Scholar, Wiley online library, Sci‐Hub and ScienceDirect. Using key words such as *A. calamus*, phytochemistry, ethnobotany, pharmacology, cognitive enhancement information was collected from the above sites. Results: *A. calamus* possesses valuable phytochemicals having wide range of biological applications. The essential oil contains high levels of *β* ‐ asarone, camphene, *β* ocimene, camphor, calarene, α selinene, and cadinol. Pharmacologically, *A. calamus* possesses diverse medicinal property including antimicrobial, antioxidant, anti‐inflammatory, anti‐diabetic potential which make the plant more valuable. Previously reported numerous studies related to *in vitro*, *in vivo* and *in silico* results showed the plant possesses significant efficacy on neurological and memory related aspects. Conclusion: The present review covers all the previous studies on neurological aspects as well as cognitive related studies.

## Graphical abstract

*Acorus calamus* is an important medicinal plants having a wide range of biological properties. Traditionally, the plant is commonly used to treat various diseases including fever, cold, pain and swelling, stomach problem, etc. The major compound found in the plant is β‐ asarone. In the present review, neurological properties including cognitive and memory related studies were reviewed with updated information. Traditionally, the plant is widely used in memory related treatment but scientific validation with clinical trial is utmost necessary for congnitive related drug development near future.

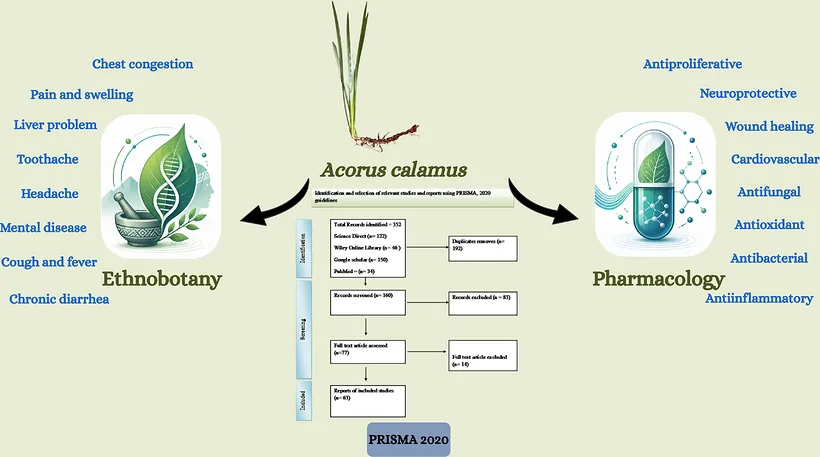

## 1 Introduction

Traditional medical systems have utilized a wide range of plants to manage cognitive disorders, including neurodegenerative conditions like Alzheimer's disease (AD) and multiple memory associated disorders [1]. To treat any pain and diseases human being have long discovered the significance of medicinal plants [2]. The valuable information regarding phytotherapy of medicinal species was written thousands of years ago. For the enhancement of memory, cognitive ability and learning there is a growing interest of studying plants phytochemical [3]. Recent prospective research on flavonoid consumption has offered compelling evidence that dietary flavonoid intake can preserve cognitive performance with ageing [4].

In recent years, growing attention has been directed toward the potential of plants and herbs to enhance memory and influence the nervous system. Plants generate a wide array of complex and biologically active compounds. These unique substances often possess neuroprotective, antioxidant and anti‐inflammatory activity typically with little to no adverse side effects [5]. These naturally occurring compounds have long been acknowledged for their ability to enhance cognitive function, support brain health, and potentially reduce memory decline associated with aging. Medicinal plants have been effectively utilized by humans to manage a range of neurological conditions, including dementia, amnesia, and AD. Moreover, research has shown that these plants may help decrease amyloid protein accumulation in the brain and reduce oxidative stress, contributing to the recovery of memory function [6].

Among the vast array of medicinal plants, *Acorus calamus* has held a prominent place in the Ayurveda since ancient times. *A calamus* commonly known as “sweet flag” in English and Vacha in Hindi language is a semi aquatic, perennial medicinal plant of Acoraceae family [7]. In Ayurveda, this plant species been widely used in various herbal traditions as a brain tonic to support the nervous system and improve memory and cognitive function [8]. This species is an essential part of Chinese and Indian medicine having long history of its applications [9]. In Charaka Samhita *A. calamus* is used in the formulation of “Yavasarshapadi Choorna” due to its medicinal properties. The species is also used to prevent infection in surgical areas and fumigation of clothes. It is recognized for its rejuvenating effects on the nervous system and brain. Charaka highlights several medicinal properties of *A. calamus*, such as promoting longevity, restoring consciousness, relieving saturation, eliminating excess body fat, treating skin conditions, alleviating hemorrhoids, and being beneficial in nasal therapies [8, 10]. In Siddha medicine, it is noted for its pediatric applications. In rural parts of southern India, the rhizome paste is traditionally used to enhance speech and memory in children [11]. This plant is commonly utilized in traditional folk medicine in both Indonesia and America to treat gastrointestinal issues such as colic pain, diarrhea, and diabetes [12]. Traditionally in India the plant species is used to treat skin diseases, stomach ache, central nervous system, fever, cough, diarrhea, asthma, kidney, and liver problems [13].

Beyond these ethnomedicinal uses, *A. calamus* in recent times is more esteemed for its cognitive‐boosting attributes, with research indicating its efficacy in enhancing memory, learning capacity, and mental attentiveness. Such effects are mostly ascribed to its bioactive components, notably asarones, which function through mechanisms such as acetylcholinesterase inhibition, oxidative stress reduction, and neurotransmission regulation. These pharmacological discoveries highlight the potential of *A. calamus* in addressing neurological health, giving a solid basis for researching its involvement in neurodegenerative disorders such as AD. AD is a long‐term, progressive neurodegenerative disorder of the brain that is related with memory loss, learning difficulties, and is the leading cause of progressive dementia in older adults [14, 15]. AD is a gradually worsening neurological condition that affects approximately 15 million people globally. It is the most common cause of dementia in older adults, with an average course of around 8.5 years from the appearance of symptoms to death [16]. Therefore, we have reviewed all research articles to highlight the study of *A. calamus* till date on central nervous system (CNS) and AD diseases and its possible research gap.

Previously, a number of studies were reported on cognitive and neurological aspects through *in vitro*, *in vivo*, and *in silico* approaches [17, 18, 19, 20, 21, 22]. Most urgently, there is need of extensive research on clinical trial and rigorous research on *β*‐asarone and its impact on human body to validate long practice ethnobotanical applications of *A. calamus*. The current review provides comprehensive information regarding phytochemical profiling, ethnobotanical practices, and recent pharmacological applications with special emphasis on memory and cognitive enhancement, which has not been jointly address in earlier reports with updated information. Besides these, the current article focuses on biology and detail toxicological information as well as *in vitro*, *in vivo*, and *in silico* experiments, which will be helpful for the researchers and people from the related field to explore more about the species aiming the development of cognitive enhancement drugs near future.

## 2 Materials and Methodology

According to PRISMA 2020 guidelines [23] the review methodology was applied for the systematic reviews and meta‐analyses.

### 2.1 Eligibility Criteria

Original research and review articles from peer review journals were used for the present review. Book chapter and valid websites were also used for the extraction of necessary information. Here, proceedings editorials and commentaries and information from conference were excluded. The search was restricted to articles published in English between 1967 and 2026 that were relevant to the scope of the present review title. Research results from animal studies, *in vitro* and *in silico* investigation were added.

### 2.2 Data Sources and Search Strategy

Key words such as *A. calamus*, phytochemistry, ethnobotany, pharmacology, cognitive enhancement and toxicology were used for searching information related to present review. PubMed, Google Scholar, SciHub, ResearchGate, Wiley Online library and National Center for Biotechnology Information were used for searching the data related to the current systematic review.

### 2.3 Extraction and Data Collection

Screening of the articles were performed based on titles and on the basis of abstract information provided in the research and review papers, irrelevant articles were excluded. All eligible articles were then carefully reviewed and analyzed. The ethnobotanical and pharmacological part related to CNS and cognitive studies were summarized in the table.

### 2.4 Analysis of Data and Reporting

The main body of the current review was constructed using the relevant information for the selected review and research articles. Figure 1 (using PRISMA, 2020 guidelines) displayed the search strategy and list of review sources that were assessed for the current study.

**FIGURE 1:**
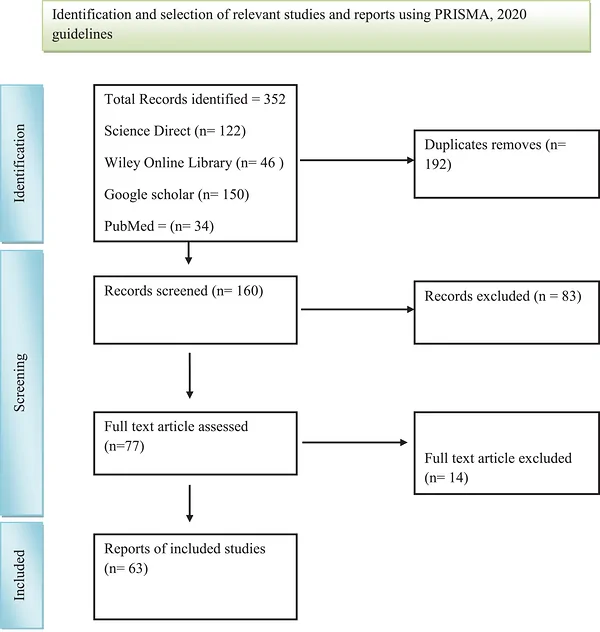
Screening and selection of pertinent studies and reports in accordance with PRISMA 2020 guidelines.

## 3 Botanical Description of *A. calamus*

*Acorus calamus* is a wetland monocot, perennial species which belongs to the Acoraceae family that grows upto 4 feet tall. The leaves of *A. calamus* feature a prominent central midvein, accompanied by slightly elevated secondary veins on either side or numerous fine tertiary veins, distinguishing it clearly from other Acorus species. Leaf widths range from 0.7 to 1.7 cm, averaging around 1 cm. The sympodial leaf is typically shorter than the vegetative leaves, and the leaf margins are wavy or curled [24]. The aromatic rhizome of *A. calamus* is thick up to 2.5 cm. The rhizome is light brown to pale green or pinkish or purplish‐brown color externally and white internally [24, 25]. The rhizome has numerous roots with brown to white color. Both the rhizome and leaves of *A. calamus* has a citrus like pleasant aroma, however, it has a sour taste. The flowers are greenish‐brown in color and cylindrical in shape and having length between 3 to 8 cm, covered with numerous rounded protrusions. The spadix it can grow up to a length of 4.9 to 8.9 cm. The fruits are small, resemble berries, and contain only a few seeds [24]. The detail botanical parts of *A. calamus* were mentioned in the Figure 2.

**FIGURE 2:**
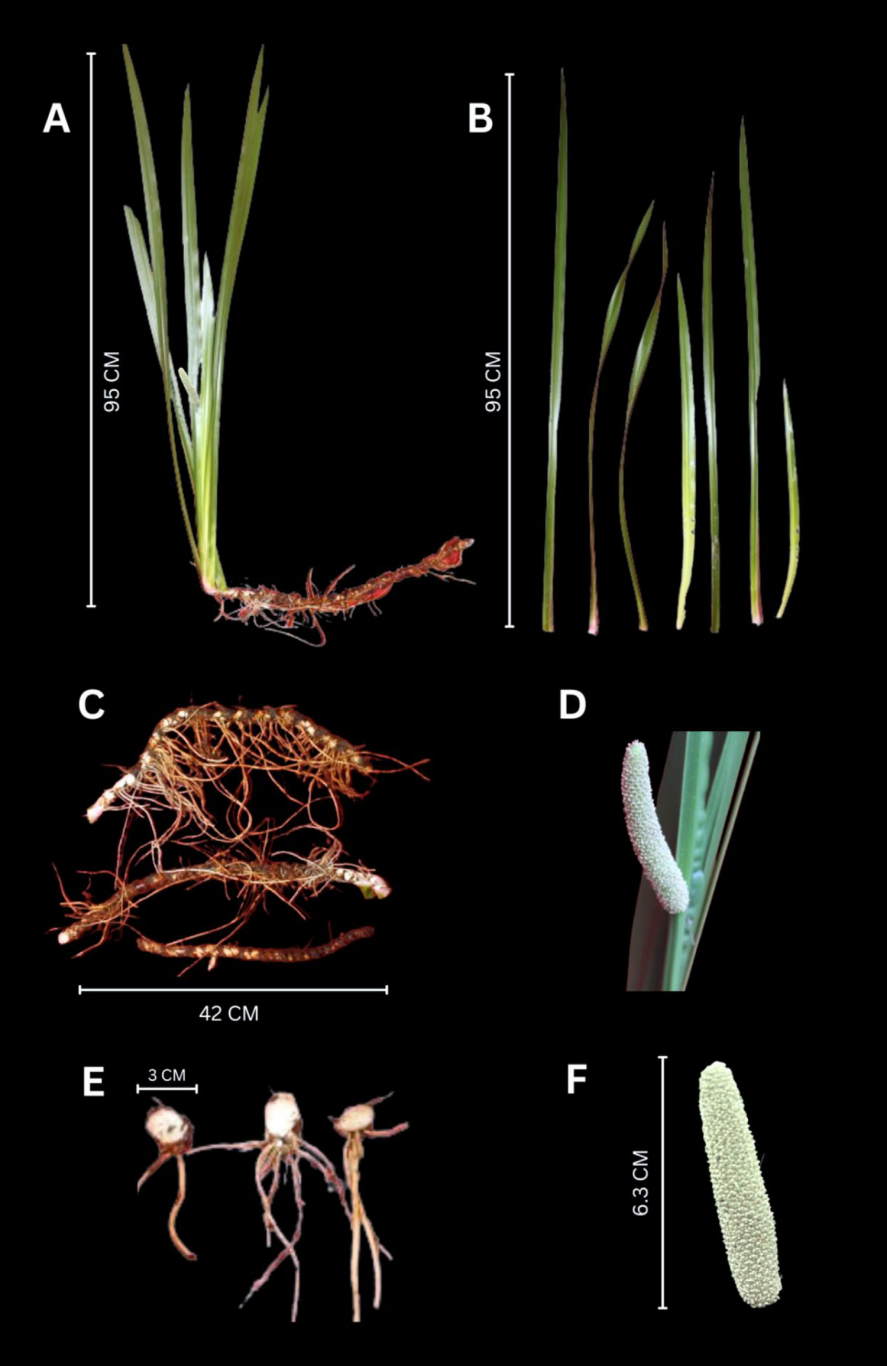
Botanical description of *A. calamus* L. (A) Habit, (B) leaves with different heights, (C) rhizome with hairy roots, (D) inflorescence with peduncle, (E) cross section of rhizome, (F) spadix.

Botanical microscopic description and pharmacological study of *A. calamus*, study of transverse section of leaves showed the xylem vessels, thick‐walled parenchyma, phloem, epidermis, aerenchyma, and vascular bundle [26]. Another morphological study of triploid and diploid variety of *A. calamus* from southern western Siberia was reported. All triploids of *A. calamus* are sterile with a uniform parthenocarpic fruits. The size of the fruits depends on developing seeds. The key difference between diploid and triploids was found as air lacunae in leaves, determined by cell number rather than cell size [27].

*Acorus calamus* rhizome possesses distinct morphological features helpful for its identification. In transverse part, it comprises of a single layered epidermis encircled with thin layer of cuticle and having a broad parenchymatous cortex. These cortical cells contain essential oil droplets with thin walled and abundant starch granules. In cortex, several schizogenous secretory cavities were present, describing a key characteristic feature of the species [26, 28, 29, 31]. The cortex is well separated by endodermis from the central stele. The vascular system is formed by the collateral, scattered, and closed vascular bundles. Phloem is composed of parenchyma and sieve elements, whereas xylem comprise of spiral and annular vessels [28, 29, 30, 31].

## 4 Ethnobotany of *Acorus calamus*

*Acorus calamus* L. has a prominent role in traditional medicinal systems such as Unani, Ayurveda, and Traditional Chinese Medicine [31, 32]. During the Vedic period, it was utilized as a ‘rejuvenator’ for the brain and nervous system. In Charaka it is classified as lekhaniya (natural substances that eliminate body fat), truptighna (anti‐saturative), asthapanopaga (an adjunct to decoction enemas), sitaprasamana (alleviates cold sensations on the skin), sanjnasthapana (restores consciousness), vayasthapana (promotes longevity), arshoghna (anti‐hemorrhoidal), and shirovirechana [10]. In Ayurvedic system, the rhizome part of the plant was used as bitter tonic, laxative, carminative, aphrodiasic, stimulant, anthelmintic properties and in curation of dysentery, cough, intermittent fevers, chronic diarrhea, asthma, and abdominal tumors [33]. The Sushruta Samhita states that the rhizome preparations of this medicinal plant species can be used to cure heart conditions, asthma, and pustular eruptions, while the juice is used to promote lifespan and intellectual vigor. In Unani literature, it is mentioned for beneficial as a brain tonic and for general debility. This is also listed in the Charaka Samhita as a resuscitative (Sanjnasthapana) [34].

*Acorus* was formerly classified inside the family Araceae; however, it is now categorized in its own family, Acoraceae, within the order Acorales, and is considered part of the oldest surviving lineage of monocotyledons. The name *Acorus* comes from the Greek word acoron, which was used by Dioscorides. It was derived from coreon, which means pupil, and was used in herbal therapy to cure eye inflammation [35]. Due to the presence of aromatic compounds in *A. calamus*, it is widely used in wellness cosmetic purposes [36].

To reduce pain and swelling in rheumatism and rheumatic fever patients, the rhizome paste of *A. calamus* is given topically to the swollen joints. Moreover, *A. calamus* is used in the treatment of piles, tinnitus, migraine, earaches, headaches, heaviness, and epilepsy [37]. In China and India, the rhizome of *A. calamus* has been used for at least two millennia where Chinese used it reduce swelling and constipation [13]. In India, rhizomes are commonly used to treat conditions such as epilepsy, mental disease, chronic diarrhoea, dysentery, fever, abdominal tumors, kidney and liver problem, and rheumatism. In China, the rhizome was used in traditional knowledge, ethno medicinal, and foods [35]. Remarkably, *A. calamus* rhizome paste has been utilized to treat speech impairments and enhance children's memory, particularly in southern India's rural communities [37]. Among the American natives, it was used as anaesthesia for headache and toothache. Besides, the rhizome part was extensively prevalent in traditional remedies among the ancient Greek and many other European cultures [13]. Its aromatic characteristics make it a popular choice for incense and potpourri. Its perfume is considered to provide calming and uplifting effects [36]. In several Asian nations, such as Nepal, locals alleviate coughs by chewing *A. calamus* rhizomes [35]. In some Indian communities, such as Udhampur district in Jammu and Kashmir, dried rhizomes powder of *A. calamus* are given orally with milk to expel intestinal worms. The rhizome paste has been shown to eradicate external parasites and is thought to strengthen teeth and relieve toothache. In Manipur, the fresh rhizomes of *A. calamus* are used to treat cough and chest congestion, particularly in newborns to prevent minor cough and fever by hanging it around their necks [37] (Figure 3).

**FIGURE 3:**
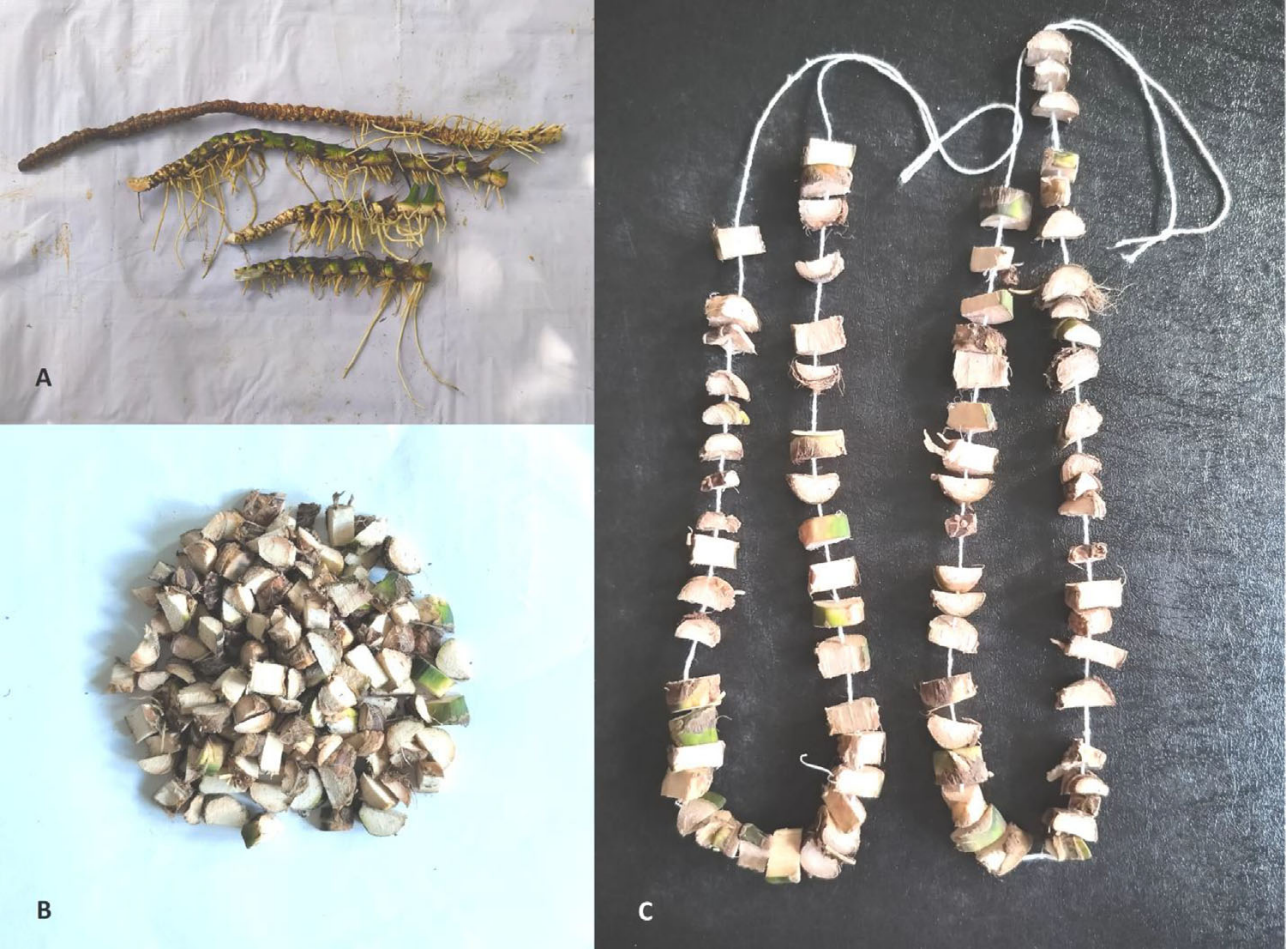
Economically important parts of *A. calamus* (A) Fresh rhizome of *A. calamus*, (B) smaller cross sections of *A. calamus* rhizome, (C) Garlands prepared using *A. calamus* rhizome, traditionally used to treat cough, cold and fever in child.

As *A. calamus* has been widely acknowledged since ancient times for its diverse ethnobotanical applications, reflecting its deep cultural and therapeutic significance over the years, scientific investigations have gradually inclined towards unveiling the key phytoconstituents responsible for its broad spectrum and validate the traditional knowledge specially cognitive and neurological related problems.

## 5 Historical Identification of *A. calamus*

*A. calamus* was reported to have been originally identified in Frankfurt in 1592 and in the dispensatorium Noricum in 1589 [38, 39]. Since 1957, this plant has been the subject of continuing phytochemical research [28]. Acknowledging the profound cultural and therapeutic significance of *A. calamus* in different subjects highlights the necessity for conservation and additional scientific confirmation to fully investigate its potential in contemporary medicine. For this, investigation has been done extensively by researchers in exploring the phytochemicals present in the *A. calamus* species in scientific literature.

## 6 Ploidy Levels in *A. calamus*

Despite the fact that *A. calamus* has a number of medicinal, aromatic, and industrial qualities, its use in food formulations and other therapeutic preparations has been prohibited by the Scientific Committee on Food and Flavoring Substances and the CEFS (Council of European Committee of Experts on Flavoring Substances) because of the plants' high levels of *β*‐asarone, which are carcinogenic and toxic. In India, it has been proposed that only types with low levels of *β*‐asarone should be used medicinally [40]. It is reported that higher *β*‐asarone content is found in tetraploids than triploids and very less in diploid genotypes. In Indian subcontinent triploid genotypes are mostly found with high *β*‐asarone contents [38].

## 7 Phytochemistry and Applications of *A. calamus*

The *A. calamus* oil is a unique brownish‐yellow essential oil, derived from various portions of the plant which is traditionally valued for its medicinal and aromatic properties. The oil has distinctive sweet smell from which the name got derived as “sweet flag”. Among the various phytochemical compounds found in the oil, the phenolic ether asarone stands out as the most bioactive ingredient, accounting for up to 96% of its makeup. Asarone comes in three different forms namely, *α, β*, and γ‐asarone among which *β*‐asarone is thought to be the oil's most distinctive ingredient. Its concentration often varies according on the plant's ploidy level, growth stage, and geographic location [38, 40].

*Acorus calamus* with higher ploidy levels had higher percentage of *β* asarone (11%), camphene (2.27%), enriched (E) *β* ocimene (3.28%), camphor (1.54%), calarene (1.42%), *α* selinene (5.02%), and cadinol (2.00%) compared to the diploid *A. calamus*. The later is constituted of higher levels of isoshyobunone (8.62%), sesquiphellandrene (3.28%), preisocalamendiol (22.81%), and acorone (26.33%). Phenylpropanoids, sterols, triterpene glycosides, triterpenoid saponins, sesquiterpenoids, monoterpenes, and alkaloids are among the 145 chemicals that have been identified approximately till date from the rhizomes and leaves of *A. calamus* [41]. Sesquiterpenoids and phenylpropanoids, primarily asarone and eugenol have been identified as the main active ingredients in *A. calamus* [28]. Another report mentioned that over 234 chemical compounds have been found till date, including phenylpropanoids, lignans, terpenoids, alkaloids, and flavonoids [42]. The oil additionally comprises fatty acids, including heptylic acid, an ester of butyric acid, palmitic acid, and its ester. Previously, a lignin called as acoradin was isolated from the rhizomatous portion of the plant species [43]. Ethnobotanical surveys and pharmacological assessments have corroborated these applications, linking the biological activities to principal phytoconstituents including α‐asarone and β‐asarone [44, 45]. Many of its traditional uses are supported by contemporary preclinical research, especially its neuroprotective, antibacterial, antioxidant, and wound‐healing qualities [46, 47]. Modern studies mentioned that essential oil is the main key component responsible for therapeutic benefits such as cell‐protective, hypolipidemic, antibacterial, anti‐inflammatory, antiproliferative, diuretic, and antiurolithic qualities. It also reduces cardiovascular and cerebrovascular problems [48]. At a lesser level, the essential oil from the rhizomes is also utilized in foods like frozen desserts, yoghurts, cakes, and confections. It is also utilized in the manufacturing of beer and alcoholic beverages like bitters, cordials, and vermouths [38]. The group of chemical classes found in *A. calamus* was listed below:

### 7.1 Phenylpropanoids

With structurally various group of phenylalanine‐derived secondary plant metabolites (C6‐C3), phenylpropanoids includes chemical compounds such as eugenol, isoeugenol, *α*‐and *β*‐asarone, ferulic acid, cinnamaldehyde, coumarin, and so on. From the *A. calamus* numerous phenylpropanoids have been detected. The major compounds identified with high percentage were *α*‐and *β*‐asarone [18, 26, 27, 49, 50, 51, 52, 53, 54, 55, 56, 57, 58, 59].

### 7.2 Monoterpenes

Monoterpenes compromises of various functional groups including phenols, alcohols, esters, ethers, aldehydes, ketones, and so on. These are simplest class of terpenes which belongs two isoprene *viz*. terpenes and terpenoids groups. Due to these organic compounds *A. calamus* leaves and rhizomes possesses fragrance having wide range of pharmaceutical applications [49, 50, 51, 52, 53, 55, 56, 57, 58, 59, 61, 62].

### 7.3 Sesquiterpenoids

A number of different sesquiterpenoids were identified in *A. calamus* including caryophyllene, isoshyobunone, cubebol, shyobunone, humulene, tau‐muurolol, and so on [57, 62] responsible for distinguish aroma of the plant as well as medicinal effectiveness [49, 53, 55, 56, 57, 58, 61, 62].

### 7.4 Triterpenoid Saponins

Triterpenoid saponins are comprised of pentacyclic C‐30 terpene. Using NMR, two triterpenoid saponins viz., 1β,2α,3β, 19α‐Tetrahydroxyurs‐12‐en‐28‐oic cid‐28‐O‐ {(β‐D‐glucopyranosyl (1‐2)}‐β‐D galactopyranoside, 3‐β, 22‐α‐24,29‐tetrahydroxyolean‐12‐en‐3‐O‐(β‐Darabinosyl (1,3)}‐β‐D‐arabinopyranoside Were Identified in *A. calamus* rhizome [63].

### 7.5 Miscellaneous Compounds

Various miscellaneous compounds such as 4,5,8‐trimethoxy‐xanthone‐2‐*O*‐*β*‐D‐glucopyranosyl (1‐2)‐*O*‐*β*‐D‐galactopyranoside, Pyrimidin‐2‐one,4‐[N‐methylureido]‐1‐[4methyl amino carbonloxy methy], Trimethoxyamphetamine,2,3,5, *β*‐daucosterol, 22‐[(6‐deoxy‐*α*‐L‐rhamnopyranosyl) oxy]‐3,23‐dihydroxy‐, methyl ester, (3*β*,4*β*,20*α*,22*β*), glutamic acid, threonine, *α*‐alanine, aspartic acid, asparagine, phenylalanine, tryptophan, lysine, proline, norvaline, tyrosin, palmitic acid, myristic acid, stearic acid, palmitoleic acid were detected [63, 64, 65, 66, 67, 68]. The details on extraction technique, phytochemical class, marker compounds, instrument applied, plant part and quantification methods were provided in the Table 1.

**TABLE 1:** The details on extraction technique, phytochemical class, marker compounds, instrument applied, plant part and quantification methods.

Plant part	Extraction technique	Phytochemical class	Key compounds	Identification technique	Instrument applied	Quantification method	References
Rhizome	Hydro‐distillation, Soxhlet	Phenylpropanoids	*α*‐asarone, *β*‐asarone	GC–MS, HPLC	GC–MS, HPLC	External standard calibration	[28]
Leaf, rhizome and roots	Hydro‐distillation	Mono‐ and Sesquiterpenes	Acorenone, α‐pinene (In rhizome); Geranyl acetate, (Z)‐β‐farnesene (Leaves); acorenone, cyclocolorenone (root)	GC–MS	GC–MS	Peak area normalization	[55]
Rhizome	Hydro‐distillation	Phenylpropanoids	*β*‐asarone, α‐asarone	GC–MS	GC–MS	Peak area normalization	[69]
Essential oil	Hydro‐distillation	Phenylpropanoids	*β*‐asarone, calerene	GC–MS	GC–MS	Peak area normalization	[62]
Essential oil	Hydro‐distillation	Phenylpropanoids	*β‐*asarone, calerene	GC–MS	GC–MS	Peak area normalization	[61]
Rhizome	Solvent extraction	Phenylpropanoids	*β*‐asarone, shyobunone;	GC–MS	GC–MS	Peak area normalization	[56]
Essential oil	Hydro‐distillation	Phenylpropanoids	*β*‐asarone, α – asarone, β ‐methyl isoeugenol	GC–MS	GC–MS	Peak area normalization	[57]
Essential oil	Hydro‐distillation	Phenylpropanoids	β‐asarone, isoshyobunone	GC–MS	GC–MS	Peak area normalization	[70]
Leaves and rhizome Essential oil	Hydro‐distillation	Phenylpropanoids, monoterpinoids	*α* ‐asarone and‐ *β* asarone in rhizome and *β* asarone and Linalool in leaf	GC–MS	GC–MS	Peak area normalization	[58]
Rhizome	Hydro‐distillation	Phenylpropanoids,	α‐asarone. (E)‐methylisoeugenol, methyleugenol	GC‐FID, GC–MS	GC‐FID, GC–MS	Peak area normalization	[53]
Rhizome	Hydro‐distillation	Phenylpropanoids,	Isoshyobunone, β‐asarone	GC‐FID, GC–MS	GC‐FID, GC–MS	Peak area normalization	[71]
Rhizome	Hydro‐distillation	Phenylpropanoids,	*α*‐asarone, *β*‐asarone	GC–MS	GC–MS	Peak area normalization	[72]
Rhizome	Hydro‐distillation	Phenylpropanoids,	Methyl isoeugenol, cyclohexanone	GC–MS	GC–MS	Peak area normalization	[73]
Rhizome	Hydro‐distillation	Phenylpropanoids,	*β*‐asarone, cis‐beta‐terpineol	GC–MS	GC–MS	Peak area normalization	[74]
Rhizome and leaves	Hydrodistillation (HD) and micro‐steam distillation‐solid‐phase microextraction (MSD‐SPME),	Phenylpropanoids, sesquiterpene	*β*‐asarone, acorenone	GC–MS	GC–MS	Peak area normalization	[75]

## 8 Previous Research on *A. calamus* L. for Cognitive Enhancement

Prior investigations into *A. calamus* L. for cognitive enhancement have demonstrated its promising neuropharmacological potential. Bioactive compounds, specifically *α*‐ and *β*‐asarone have demonstrated the potential to regulate cholinergic neurotransmission, decrease acetylcholinesterase activity, and augment synaptic plasticity, thereby enhancing learning and memory functions. Previously, Mukherjee et al., [8] studied the acetylcholinesterase (AChE) inhibitory activity of essential oil and hydraulic extract of *A. calamus* using standard protocol of Ellman's. The IC_50_ values were obtained for the *A. calamus* essential oil (10.67 ± 0.81 µg/mL), hydroalcoholic extract (182.31 ± 16.78 µg/mL) as well for the standards of of β‐asarone (3.33 ± 0.02 µM/mL) and α‐asarone (46.38 ± 2.69 µM/mL) were assessed. Physostigmine used as the standard control exhibited an IC_50_ value of 0.28 ± 0.015 µM.

The reported IC_50_ value of *β*‐asarone (3.33 µM) indicates moderate inhibitory activity against acetylcholinesterase when compared to the reference drug physostigmine (0.28 µM), reflecting approximately 10–12‐fold lower potency. While this suggests that *β*‐asarone is less potent as a direct enzyme inhibitor, such differences are not uncommon for natural compounds. Importantly, the overall pharmacological effect may still be relevant due to multi‐target activity, including antioxidant and anti‐inflammatory effects. Though β‐asarone revealed moderate effectiveness compared to standard drug physostigmine, it has pharmacological importance since it is plant‐based compound and can contribute therapeutic activity. This study concludes that major phytoconstituents of *A. calamus* that is β‐asarone showed moderate inhibitory potential along with *A. calamus* essential oil showing significant AChE activity. In this study, due to the limited selectivity data particularly on inhibition of butyrylcholinesterase (BuChE), confines the detail understanding of action mechanism. Besides these, the significance results of this data need further validation through in vivo and clinical experiments. However, further studies evaluating selectivity, binding affinity are required to determine its therapeutic potential.

Another study reported the effects of central nervous system on animal model (rats and mice) using ethanol extract of *A. calamus* rhizome [76]. They have studied multiple experiments on animal model including potentiation additive of sleeping time, effects on conditioned avoidance response, effects on spontaneous motor activity, hypothermia screening, behavioral studies, anticonvulsant evaluation, analgesic evaluation, effects on adrenal ascorbic acid. *A. calamus* (0.5 mL/kg, i.p.) potentiated the prolongation of pentobarbitone‐induced sleeping time in rats. Additionally, an increase in the duration of loss of the righting reflex was observed in mice when administered with ethanol and ether. *A. calamus* (AC‐1, 0.5–1.0 mL/kg, i.p.) exhibited notable CNS depressant properties. It significantly reduced body temperature in rats, with an effect similar to chlorpromazine, and strongly suppressed food‐seeking behavior in fasted rats, indicating potent sedative or antipsychotic‐like activity. However, it failed to reduce apomorphine‐induced aggression, suggesting limited dopamine receptor antagonism. *A. calamus* showed no analgesic effects in chemical, mechanical, or thermal pain models. In seizure studies, it offered moderate protection against pentylenetetrazol (PTZ)‐induced convulsions, reducing severity and mortality, but showed no efficacy against strychnine‐ or electroshock‐induced seizures, indicating a narrow‐spectrum anticonvulsant action, possibly through GABAergic mechanisms [76]. *A. calamus* leaf extract exhibited dose‐dependent effects on exploratory behavior, locomotion, movement coordination and muscle tone of the experimental animals. In contrast, the reference drug Bilobil (100 mg/kg) had no significant impact on behavior, depressive symptoms, anesthesia response, or physical endurance in mice [77]. Previous study reported on the efficacy of rhizome extract of *A. calamus* on anxiety, memory loss, oxidative stress and lipopolysaccharide induced neuro inflammation using rat models [78]. Animal treated with various fractions of *A. calamus* showed improved performance in the shuttle box test compared to the lipopolysaccharide (LPS) control group. In the elevated plus‐maze (EPM) test, these groups also exhibited reduced stress levels relative to the LPS group. The aqueous fraction at a dose of 600 mg/kg demonstrated the most significant improvement in memory and the lowest stress levels. Overall, the findings suggest that various fractions of *A. calamus* exert dose‐dependent protective effects against memory and stress problems, likely by modulating oxidative stress and inflammation [78].

A study was conducted on the effect of α‐asarone on hippocampal dependent memory in noise stress exposed rats. They have investigated that whether, exposure of noise for 100 dBA/4 h/d for 30 days could damage memory loss in rats and whether, α‐asarone (9 mg kg‐1 b.wt.) and ethyl acetate extract of *A. calamus* (AC EAAC) 50 mg kg‐1 b.wt.) can prevent the memory loss due to exposure of noise. Along with that AChE and antioxidant activity was assessed in hippocampus after rats were examined in Radial Eight‐arm Maze (RAM). It was found that α‐asarone and AC EAAC prevents the noise stress affect induced memory problems in studied animals [79]. Another study reported on effects of rhizome extract of *A. calamus* on neuromodulatory system in restraint stress male rats. Male rats in the stress group had significantly lower sodium potassium ATPase activity and TBARS levels than the control group. Following administration of *A. calamus* rhizome extract, these parameters tended to return to normal. The *A. calamus* rhizome powder demonstrated modulatory influence on antioxidants and Na‐K‐ATPase activity for stress‐induced cognitive processes [18]. Similarly, another research was conducted using in vitro activity of extract of *A. calamus* and α‐asarone on oxidative stress‐ and endoplasmic reticulum (ER) stress induced cell death in hippocampal HT22 cells. Both α‐asarone and *A. calamus* extract considerably reduced cell mortality brought on by the ER stress inducer tunicamycin and the oxidative stress inducer l‐glutamate. These results suggested that AD and other neurodegenerative disorders may be effectively treated with α‐asarone [19]. The effects of α‐asarone on memory disorders on rat against scopolamine induced amnesia were assessed. A dose of 1 mg/kg body weight of α‐asarone was administrated for 14 days to rats which resulted in improvement of scopolamine‐induced memory impairment. Besides these, it was found that, α‐asarone treatment significantly (*p* <0.05) decreases the brain acetylcholinesterase activity in hippocampus, cortex and striatum brain regions of amnesic rats. The results suggested that the α‐asarone possesses anti‐cholinesterase and anti‐amnesic effectiveness and can be applied for the treatment of AD [20]. Neuroprotective and antioxidant effectiveness of *Cordia dichotoma* and *A. calamus* in Alzheimer's type dementia in rodent was studied. *A. calamus* extract alone was used orally in the mice using hydro‐alcoholic 1:1 with 600 mg/kg and the combination of this with *Cordia dichotoma* 750 mg/kg orally. The combinative dose of *A. calamus* and *Cordia dichotoma* revealed significant results showing scopolamine‐induced Alzheimer's type dementia in mice by activating the antioxidant, cholinergic system, and protection of neuronal death in the hippocampus regions of brain. Besides these, behavioral parameters *viz*., elevated plus maze (EPM), morris water maze (MWM) and locomotor activity were applied and found reduced after the exposure of Scopolamine (1 mg/kg i.p.). It decreases antioxidant levels (superoxide dismutase and reduced glutathione) but conversely increases the level of AChE in brain [22]. The biological activities of *A. calamus* were given in Table 2.

**TABLE 2:** Biological activity on *A. calamus* L. reported.

Activity	Plants parts	Methods	Extarcts/solvents/ essential oil	Dose	Outcome/results/effects	Reference
Anti‐cholinesterase activity	Rhizome extract	AChE activity of hydroxyapatite nanoparticles derived from an *A. calamus* (AC‐HAp NPs) and In silico mediated by APOE4, BNDF, γ‐secretase proteins, PKC‐γ, and BACE1	Hydroxyapatite nanoparticles from rhizome extract	6.25, 12.5, 25, 50, 100 ug/mL	Prepared AC‐HAp NPs can inhibit AChE	[21]
Anti‐cholinesterase and anti‐amnesic activity	*α*‐asarone isolated from *A. calamus*	AChE assay using Ellman's method and the anti‐amnesic activity investigated using active avoidance tests, passive avoidance elevated plus‐maze test.	α‐asarone isolated from *A. calamus*	Effects of α‐asarone against scopolamine induced amnesi was assessed using 1 mg/kg body weight.	α‐asarone revealed anti‐cholinesterase and anti‐amnesic activity	[20]
Hippocampal dependent memory in noise stress exposed rats	Extract of *A. calamus and α‐Asarone*	AChE, animal model (rat) with Radial Eight‐arm Maze (RAM	Ethylacetate extract of *A. calamus*	α‐asarone (9 mg kg‐1 b.wt.); ethyl acetate extract of *A. calamus* (AC EAAC) 50 mg kg‐1 b.wt.	α‐asarone and AC EAAC prevents the noise stress effected indured memory problems in studied animals.	[79]
Alzheimer's type dementia	*A. calamus* extract	Elevated Plus Maze (EPM), Morris Water Maze (MWM) and locomotor activity	Hydro‐alcoholic solvents with *A. calamus* and *Cordia dichotoma*	Hydro‐alcoholic 1:1 with 600 mg/kg and the combination of this with *Cordia dichotoma* 750 mg/kg orally	Decreases antioxidant levels (superoxide Dismutase and reduced glutathione) but conversely increases the level of AChE in brain.	[22]
Antihperglycemic activity	Rhizome	Oral glucose tolerance test	Methanol	200 mg/kg extract oral administered to diabetic rats for 21 days	Oral administration of methanolic extract improved blood glucose levels. After 21 days of treatment, reductions were observed in lipid profile, glucose‐6‐phosphatase, blood glucose, fructose‐1,6‐bisphosphatase, and liver enzyme markers compared to the diabetic control group.	[80]
Anti‐diabetic activity	Rhizome	Insulin‐mediated glucose consumption Model of HepG2 cells.	Ethyl alcohol	DMSO and 129 mg/mL Metformin (Met)	A novel sesquiterpenoid compound, 1β,5α‐guaiane‐4β,10α‐diol‐6‐one, demonstrated significant anti‐diabetic potential by enhancing insulin‐mediated glucose uptake in HepG2 cell models.	[81]
Antioxidant	Rhizome	DPPH, NOx free radical scavenging activity	Methanol	(50, 100, 150, 200, 250, 300) µg/mL	Strong antioxidant with an IC_50_ of 192.99 µg/ml for DPPH and 236.60 µg/ml for nitric oxide radical scavenging activity	[82]
Antidiabetic	Rhizome	Alpha‐glucosidase enzyme inhibition assay	Methanol	(25, 50, 100, 200, 400) mcg/ml	Significant alpha‐glucosidase inhibitory activity (IC50 value at 54.96 µg/ml) and alpha‐amylase inhibitory activity (IC50 45.15 µg/ml) whereas acarbose showed 30.745 µg/ml.	[82]
Antidiabetic	Root	Glucose tolerance test	Ethyl acetate	100 mg/kg, i.g. revealed hypoglycemic effects	ACE at a dose of 100 mg/kg significantly reduced fasting blood glucose levels in both STZ‐induced and db/db diabetic mice, and enhanced glucose tolerance during the OGTT in diet‐induced obese (DIO) mice.	[83]
Antioxidant	Rhizome	DPPH, reducing power	Essential oil	1.5, 3, 6, 12, 24, 48 µg/mL	The rhizome essential oil demonstrated strong antioxidant (DPPH) assay, exhibiting an IC_50_ value of 1.68 µg/mL, whereas ascorbic acid, with an IC_50_ of 1.48 µg/mL.	[62]
Antioxidant	Rhizome	DPPH, reductive ability, metal chelating activity	Methanol	50‐250 µg/mL	Sample seventh from Almora showed highest antioxidant activity. In reducing power (119.42+‐0.630 ug/ml), DPPH (143.35 +‐ 0.74 ug/ml)	[84]
Antibacterial	Rhizome	Disc‐diffusion test using MIC and MBC test	Essential oil	10 µL/disc for *A. calamus* essential oil	Notable antibacterial activity was shown against *Bacillus subtilis* and *Staphylococcus aureus* with diameters of 20.11 ± 0.28 mm and 18.06 ± 1.36 mm, respectively. *Pseudomonas aeruginosa* and *Escherichia coli* exhibited slightly smaller inhibition zones of 15.58 ± 0.68 mm and 16.00 ± 0.04 mm.	[69]

## 9 *In Silico* Studies on *A. calamus* on Cognitive and Memory Aspects

Previous study reported [85] an *in silico* investigation of *A. calamus* for its potential use in treating mental depression. Compound data were sourced from Dr. Duke's database, with protein target predictions conducted via Swiss Target Prediction and ADME profiling performed using Swiss ADME. Out of 69 compounds screened from *A. calamus*, 12 met the ADME and drug‐likeness (DL) criteria, indicating their potential as therapeutic agents. The study identified key neurotransmitter‐related pathways, particularly involving serotonin, as relevant to depression therapy. Notable target proteins included ESR1, MAOA, CRHR1, GRM5, HTR2A, SLC6A4, DRD2, and CHR. Among the active compounds showing potential antidepressant effects were *β*‐asarone, aceteugenol, acoramone, acoradin, alpha‐asarone, calamenone, camelone, elemicin, sekishone, isoacorone, *trans*‐isoelemicine, and epoxyisoacoragermacrone. The study showed 60 bioactive compounds in *A. calamus* associated with 365 protein targets. Of these, three proteins were specifically linked to depression treatment, with compounds such as acoradin, acoramone, calamenone, elemicin, isoacorone, and sekishone showing promising interactions. The findings support the potential of *A. calamus* as a candidate for further *in vivo* and *in vitro* research in the development of antidepressant therapies [85].

Another study on computational pharmacokinetics, synthesis, conceptual DFT‐based and anti‐acetylcholinesterase efficacy of hydroxyapatite nanoparticles derived from *A. calamus* extract. The predicted biological targets and the ADMET properties related to the bioavailability and computational pharmacokinetics of the five ligands have been documented. Molecular docking simulations indicated that several compounds from the rhizome extract of *A. calamus* revealed strong binding affinities with the selected targets. The findings also support the potential use of multifunctional nanoparticles with broad therapeutic effects [21]. The AChE activity of hydroxyapatite nanoparticles obtain from the rhizome extract of *A. calamus* (AC‐HAp NPs) were studied. From this study it was found that, the phytoconstituents derived from rhizome extract of *A. calamus* was mediated by PKC‐γ, BNDF, APOE4, γ‐secretase proteins and BACE1, this suggested that prepared AC‐HAp NPs can inhibit acetylcholinesterase activity [21].

## 10 Other Biological Activity with *In Silico* Studies on *A. calamus*

Bioinformatics and computational studies have become indispensible tools to understand the interaction of phytocompounds with molecular targets involved in cognitive activity. Software such as Schrodinger Glide, AutoDock Vina and AutoDock are commonly used to predict interaction and binding profiles. Numerous computational approaches have been applied to study the therapeutic effectiveness of *A. calamus* using pharmacoinformatics, molecular docking and molecular dynamics simulations.

The pharmacoinformatics study and molecular docking of bioactive compounds of *A. calamus* on antidiabetic agents were studied. Compounds from *A. calamus* L. such as a galgravin have strong binding affinity to the α‐glucosidase enzyme with a docking score of −8.40 kcal/mol.

Pharmacokinetic predictions reveal that the test compound is well‐absorbed and adheres to established drug‐likeness criteria [86]. Their research suggested that development of natural antidiabetic agents from *A. calamus* molecular dynamics simulations were performed using MOE 2022. Molecular dynamics simulations were performed using GROMACS 2023.3. PreADMET and SwissADME used for pharmacokinetic prediction. PubChem and Protein Data Bank used for ligand and receptor retrieval. The docking study showed galgravin possesses the strongest interaction with the α‐glucosidase active site through hydrogen bond interactions involving His114, Asp71, Asp348, Tyr74, and Arg214 residues. Again, tataroside formed hydrogen bonding with His347 whereas veraguensin interacted with His83. Acarbose interacted with Asp71, Glu247, and Arg440 residues within the catalytic pocket of α‐glucosidase. PMSF analysis exhibited minimal residue fluctuations within the active site region especially Tyr74, Asp348, Asp71, Arg214, and His114, authenticating stable ligand formation throughout the simulation time. The RMSD trajectory of the galgravin–α‐glucosidase complex remained constant throughout the 100 ns simulation (average RMSD fluctuation between 1.8 and 2.4 Å), suggesting stable protein–ligand interactions [86].

Five largest phytochemicals found in the *A. calamus* extracts were acorone, acoric acid, acoradin, calamendiol, and acoronene. Molecular docking results revealed five compounds bind to the Pro291, Arg599, Lys388, Pro431, and His487 of the dengue virus NS3 protease‐helicase, the ligand‐binding site that plays an important function in viral replication. The lowest binding energy of −299.7 kcal/mol was showed by acoradin‐NS3 protease‐helicase complex. Form the results it can be concluded as *A. calamus* extract may have prospects as a drug for dengue virus infection [87]. Docking study revealed principal amino acid residues associated with ligand binding included Pro291, Arg599, Ala606, Asp409, Lys388, Pro431, Arg387, Val544, Glu490, Ile365, and His487. Hydrophobic interactions and hydrogen bonding were mainly responsible for stabilization of the ligand–protein complexes. For the validation of docking results, molecular dynamics simulations were analyzed using GROMACS 2023.3. RMSF analysis revealed comparably lower fluctuations in the catalytic site residues including Lys388, Arg599, His487, and Pro431 suggesting persistent interactions during the simulation time. The RMSD trajectories suggested the protein–ligand complexes showed structurally stable performances throughout the 100 ns simulation time. The RMSD values (1.8‐2.5 Å) indicated minimal deviation and stable ligand accommodation within the active site [87].

Another report studied the antibacterial efficacy of *A. calamus* against *Helicobacter pylori* using molecular docking [88]. From the Protein Data Bank (PDB), the target proteins were retrieved. Protein validation was performed using the PROCHECK and UCLA‐DOE LAB SAVES v6.0 server to determine the percentage of residues present in the allowed regions of the Ramachandran plot. Using PyRx 0.8 software, molecular docking studies were carried. Through BIOVIA Discovery Studio Visualizer, interaction visualization and analysis were performed. The molecular docking analysis suggested the phytocompounds interacted strongly with several active site residues including SER A:7, TYR A:85, GLU A:113, HIS A:12, ARG A:40, LYS A:36, ARG B:50, ARG B:128, LYS B:154, GLU B:54, SER B:53, and GLY B:93. At RMSF analysis, in the catalytic site residues comprising ARG B:128, LYS A:36, LYS B:154, and ARG B:50 showed lesser fluctuations, indicating constant ligand interactions during the simulation. Among the studied phytocompounds of *A. calamus*, 2,3,5‐trimethoxyamphetamine and shyobunone revealed higher interaction stability and binding affinity against TOXB and LUXS proteins. The RMSD and RMSF and lower binding energy outline suggested strong inhibitory effectiveness of these compounds against the target proteins of *Helicobacter pylori* [88].

The antimicrobial efficacy of *A. calamus* using computational approach was studied. Among the selected phytoconstituents of *A. calamus beta* cadinene revealed best binding interaction in complex with fungal (1EA1) protein targets and bacterial (6VJE) with binding energy of 7.73 ± 0.15 kcal mol 1 and 7.66 ± 0.1, respectively. The molecular dynamics simulation study demonstrated that *β‐*cadinene effectively occupied the binding pocket of both fungal and bacterial targets and remained stable throughout the simulation period. Based on the findings of *in silico* study, b‐cadinene, a key phytocompound found in *A. calamus* is suggested as a potential candidate for the treatment of bacterial and fungal infections [89].

Another study revealed the synergistic effects of *A. calamus, Bergenia cilita, and Dioscorea bulbifera* extracts on antimicrobial activity and molecular docking analysis. The result suggested that three plant extract showed more effective antimicrobial results compared to individual plant sample. Combined sample extract showed most effectual result for antibacterial assay against *P. aeruginosa, E. coli*, and *B. subtilis* compared with the individual samples [90].

Molecular docking analysis revealed that *α*‐asarone showed binding affinities of −5.7 kcal/mol with PBP2 and −5.6 kcal/mol with the 50S ribosomal protein. Conversely, *β*‐asarone exhibited an affinity of −5.6 kcal/mol for PBP2 and −5.7 kcal/mol for the 50S ribosomal protein. Both *α*‐ and *β*‐asarone demonstrated stronger binding affinities compared to the control. Docking studies indicated that these compounds formed ionic interactions with the TYR529 residue and polar interactions with the ASN552 residue of PBP2. These findings suggest that *α*‐ and *β*‐asarone possess antibacterial potential by disrupting bacterial cell wall permeability [54]. Another studied the comparative chemical screening and pesticidal activity and molecular docking of *A. calamus*. The molecular docking studies showed the mode of action of proteins of nematodes, weeds, and insects were found in support of *in vitro* experiments [91]. Another study reported the chemical screening and *in silico* analysis of *A. calamus*. This study concludes that phytochemicals of *A. calamus* may inhibit NDM‐1‐mediated resistance in *K. pneumoniae* and could be an alternative therapeutic strategy for targeting NDM‐1‐containing *K. pneumonia* [92]. Another study was performed on phytochemistry and antioxidant efficacy of *A. calamus* essential oil. Besides these, they have studied the *in silico* studies. The IC_50_ values of the rhizome essential oils for DPPH radical scavenging (22.28 to 61.96 µg/mL), metal chelation (29.55 to 159.26 µg/mL), and reducing power (21.41 to 61.19 µg/mL). The potential mechanism of action and the structure‐activity relationship between the key components of essential oils and antioxidant‐related proteins were investigated through *in sili* *co* molecular docking, and the findings were consistent with the *in vitro* results [93].

Recently, *in silico* simulation on ischemic heart disease using bioactive compounds from *A. calamus* was studied. An *in silico* investigation showed that 19 primary active compounds from *A. calamus* interact with 48 targets involved in key biological processes related to ischemic heart disease. Notably, *α‐*asarone, galangin, *β*‐asarone, and isoelemicin demonstrated strong interactions with critical targets such as cytochrome P450 1A2, prostaglandin G/H synthase 2, transcription factor p65, tyrosine‐protein kinase, and vascular endothelial growth factor A. The study also emphasized the binding relationships between these four major phytochemicals and the five most significantly enriched targets associated with the disease [94].

The anti‐inflammatory properties of *A. calamus* was investigated using *in vitro* and *in silico* approaches. Two major phytochemicals, *α*‐asarone, and monolinolein TMS were selected for molecular docking against four receptors namely 5I1B, 4COX, 1ALU, and 2AZ5. Among these, *α*‐asarone and monolinolein TMS exhibited the highest docking scores and lowest binding energies against receptors 1ALU and 4COX, respectively, indicating strong anti‐inflammatory potential. Additionally, the application of arbuscular mycorrhizal fungi (AMF) was found to enhance the medicinal value of *A. calamus* organically, with *α*‐asarone identified as the predominant compound exhibiting confirmed anti‐inflammatory effects based on docking results [95].

## 11 Toxicological Reports and Safety Considerations of *A. calamus* and *β*‐asarone

The pharmacological properties revealed by medicinal plant *A. calamus* are mainly due to the presence of marker compound *α*‐ and or *β*‐asarone. It showed various biological activities including anti‐ inflammatory, cancer prevention, and treatment of cardiovascular problems, AChE assay, anticonvulsant, antioxidant, antimicrobial [20, 21, 62, 69, 80, 81, 82, 83, 84]. The toxicological investigation of *A. calamus* has been performed by various researchers including *in vitro*, *in vivo*, and pre‐clinical methods and safety parameters have been identified. Some of significance previous research reports were provided below with safety parameters.

A study was performed on the acute and chronic toxicity of rhizome ethanolic extract of *A. calaus* in Wistar rats. In the acute toxicity study, oral dose of 175, 550, 1750, and 5000 mg/kg body weight of ethanolic extract in female rats according to OECD 425 and observed during 24 h after oral administration. In the chronic study, doses of 0, 200, 400, and 600 mg/kg body weight daily for 90 days in Wistar rats was applied. Weight, food consumption, body weight, clinical biochemistry, haematology, and histology were investigated. In pathological study, no changes observed under gross abnormalities or histopathological disturbances. Besides these results, no significant information was observed in feed consumption and body weight and recorded zero mortality. In summary*, A. calamus* ethanolic extract does not showed any acute or chronic toxicity under the investigation in Wistar rats [96]. Acute and sub acute toxicity of hydro alcoholic extracts of *A. calamus* (HAE‐AC) was studied. General behavioral abnormalities were shown in rats at the oral single administration of HAE‐AC at a doses of 2500–10 000 mg/kg. Doses of HAE‐AC are positively correlated with the mortality of the rats. After 28 days of observation, daily single oral dosages of HAE‐AC 200, 500, and 1000 mg/kg were shown to be well tolerated behaviorally and did not significantly alter the organ and body weights of the rats. Aspartate transaminase (AST), alanine transaminase (ALT) and histopatholological alterations in liver tissue were observed at 1000 mg/kg dose of HAE‐AC. This study concludes HAE‐AC does not show any toxicity, conversely at a high dose mild toxicity was observed [97].

A study was performed on the effect of *α*‐asarone in *in vitro* and *in vivo* in human lymphocytes and mouse bone marrow respectively. The effects of *α*‐asarone has been observed was slight but continuous increase in sister chromatid exchange (SCE) frequency in both murine bone marrow cells and human lymphocytes, suggesting possible damages in DNA under study [98]. A recent study using Ames test on *A. calamus* rhizome power and marker compound *β* asarone, α asarone, and shyobunone was performed. The Ames test was performed using *Salmonella typhimurium* strains such as TA97a, TA98, TA100, TA102, and TA1535 under good laboratory conditions. The study dose ranges from 39.06 to 5000 µg/plate and did not exhibit any mutagenic changes in all tested samples [99]. Animal models used in study and risk of bias (qualitative) and behavioral assay consistency in *A. calamus* were summarized in Table 4.

The *in vitro* and *in vivo* effects of *β*‐asarone in human colorectal cell lines HT29 and SW480 in MTT assays was assessed. Through senescence‐associated *β*‐galactosidase activity, cell senescence was observed. Using Western blot, expression of cancer and senescence‐related proteins, namely lamins, Oct‐1, p53, p21, and p15 were assessed. The expression of lamin B1, p53, p21 was increased by *β*‐asarone which also regulates p15 expression by regulation of Oct‐1 binding. In summary, this result highlights the potential effectiveness of *β*‐asarone which inhibits the *in vitro* and *in vivo* colon cancer by inducing senescence [100]. Another study reported the effects of rhizome extract of *A. calamus* on neuromodulatory system in restraint stress rats for 21 days and *A. calamus* was administrated orally. For the test, elevated plus maze and Hebb‐Williams maze behavioral models were used. The study revealed that *A. calamus* rhizome powder's ability to prevent stress‐induced cognitive impairments and its ability to modulate antioxidants and Na‐K‐ATPase activity. This study unequivocally shows that *A. calamus* has a neuroprotective effect against neuronal damage caused by restraint stress [18].

The effect of asarone and essential oil of *A. tatarinowii* was reported to have mild toxic effect was found in intragastric administration of 400 and 500 mg/kg asarone with increase dose of asarone, the toxicity was also increased. With 600 with 24 h exposure and 700 mg/kg with 2 h exposure the mortality of mice increases from 33.3% to 90% and a dose of 800 mg/kg for 0.5 h caused death of experimented mice which suggested that the toxicity of asarone was increased with increased doses [101]. It was found that β‐asarone is potentially carcinogenic. In this study, the rats were fed two years with mixture of β‐asarone with different concentrations. As a results, in small intestine, tumors were identified and were found in 800 ppm (0.08%) and 2000 ppm (0.2%). Also, at the concentrations of 800 and 2000 ppm (0.08 and 0.27%) thrombosis within the chambers of the heart was observed [102]. The effects of β‐asarone on duodenal cancer were studied, which inhibited the CNS and revealed hepatotoxic activity [103]. The US FDA declared the *A. calamus* essential oil banned due to the carcinogenic properties of asarone [104]. In chronic toxicity study, it was found that the α or β‐asarone induces liver cancer on experimented animal rat at 10 months with a single dose of it for 12 days (52 mg/kg, i.p.) meanwhile, 13 months for continuous doses with days 1, 8, 15, and 22 with a total dose of about 1 mg, i. p. revealed autopsy [105]. β‐asarone (GI50 40.0 ± 2.0 µg/mL) and α asarone (GI50 46.0 ± 1.0 µg/mL) were found cytotoxic to human lymphocytes (THLE‐2) cells [106].

The chemical screening of *A. calamus* along with quantitative and qualitative parameters were studied. *A. calamus* plays a vital role in Indian Ayurvedic formulations and widely used in memory and neurological disorders after Sodhana (detoxification/ purification). As mentioned in ancient Ayurvedic text Bhaisajya Ratnavali and Rasatrangini, sodhana means purification of potentially toxic plants or substances into nonpoisonous material. The presence of *β*‐asarone in large amount in *Acorus* makes it carcinogenic and genotoxic but is broadly used in Indian Ayurvedic formulations after detoxification. In this study, detoxification was performed using gomutra and after that again boiled it with alambusa kwath (*Spaeranthus indicus*) and further boiled with *panchapallav kwath* (leaves of five plants, namely; *Neolamarckia caddamba, Syzygium cumini, Saraca indica, Mangifera indica, and Ficus racemosa*). After detoxification, it was found that the content of *β*‐asarone decreased to lesser toxic form [49]. Animal models and translational relevance and dose comparison and human relevance on *A. calamus* and *β*‐asarone were summarized in the Table 3.

**TABLE 3:** Animal models and translational relevance and dose comparison and human relevance on *A. calamus* and *β*‐asarone.

Animal models and translational relevance					Dose comparison and human relevance					Reference
Animal model	Induction method	Behavioral tests	Key outcome	Translational relevance	Extract Type	Dose (mg/kg)	Route	Duration	Comment on human relevance	
Rat	Noise stress exposure (100 dBA/4 h/day for 30 days)	Radial Eight‐arm Maze (RAM)	Noise stress caused spatial memory impairment, increased in oxidative stress, AChE activity, and overexpression of Hsp70 in hippocampus	Mimics chronic environmental noise‐induced cognitive dysfunction and oxidative stress noticed in humans exposed to prolonged noise pollution	Ethyl acetate extract of *A. calamus* (EAAC) and α‐asaron	EAAC: 50 mg/kg b.wt.; α‐asarone: 9 mg/kg b.wt.	Not specified	30 days	potential antioxidant and neuroprotective activity against stress‐related cognitive impairment in humans; however, long‐term safety of α‐asarone requires careful assessment due to reported toxicity concerns	[79]
Rats and mice	CNS pharmacological screening using anticonvulsant, sedative, and behavioral models	Pentobarbitone‐induced sleeping time, conditioned avoidance response, spontaneous motor activity, hypothermia screening, food‐seeking behavior, apomorphine‐induced aggression, PTZ‐induced seizures, electroshock and strychnine seizure models, analgesic tests	CNS depressant activity, potentiated sleeping time, induced hypothermia, suppressed food‐seeking behavior, and provided moderate protection against PTZ‐induced seizures; no analgesic activity shown	Suggests sedative, anxiolytic, antipsychotic‐like, and narrow‐spectrum anticonvulsant potential relevant to neurological and neuropsychiatric disorders	Ethanol extract of *A. calamus* rhizome	0.5–1.0 mL/kg	Intraperitoneal (i.p.)	Acute experimental studies	CNS depressant and anticonvulsant applications; however, translation to humans requires caution due to limited mechanistic data and safety concerns related with α‐ and β‐asarone	[76]
Mice	Evaluation of psychotropic and neurotropic effectiveness through behavioral and CNS function models	Open field test, tail suspension test, thiopental‐induced anesthesia, forced swim test	Dose‐dependent effects on locomotor activity, exploratory behavior, anxiety, depression, muscle tone. Moderate actoprotective activity (at 1 and 5 mL/kg), analeptic activity (at 5 mL/kg)	Suggested potential application in management of CNS related problems including depression, anxiety, fatigue, and impaired motor coordination	Dealcoholized water‐alcohol extract of *A. calamus* leaves (ECL)	1 and 5 mL/kg	Intragastric	Not specified	Possible neuroprotective and psychotropic benefits; further pharmacological and toxicological studies are necessary before clinical application	[77]
Rat	Lipopolysaccharide (LPS)‐induced neuroinflammation and oxidative stress	Shuttle box test, Elevated Plus Maze (EPM)	Memory performance and reduced stress/anxiety compared to the LPS control group. The aqueous fraction at 600 mg/kg revealed the high improvement in memory and lowest stress levels	Mimics neuroinflammatory and oxidative stress‐related with cognitive impairment	Aqueous rhizome fraction	600 mg/kg (aqueous fraction most effective)	Not specified	Not specified	Suggests dose‐dependent neuroprotective, anxiolytic, and anti‐inflammatory potential of in CNS disorders; clinical safety assessment is required before human use	[78]
Wistar rats	Scopolamine‐induced amnesia (1 mg/kg, i.p.)	Elevated plus‐maze, passive avoidance task, shuttle‐box active avoidance test, brain AChE estimation	α‐asarone increased memory and learning. Reduced transfer latency, increased step‐through latency, and improved conditioned avoidance responses. Reduced AChE activity in the hippocampus, cortex, and striatum of amnesic rats	Mimics cholinergic dysfunction and cognitive impairment associated with Alzheimer's disease and supports the therapeutic potential of cholinesterase inhibition in dementia management	α‐asarone isolated from *A. calamus*	15 and 30 mg/kg	Intraperitoneal (i.p.)	14 days	Demonstrates anti‐amnesic and anti‐cholinesterase activity with strong application in Alzheimer's disease management; α‐asarone may enhance acetylcholine levels and cognitive function, further clinical safety and toxicity studies are required	[20]
Adult Wistar male rats	Restraint stress for 21 days (6 h/day) inducing cognitive dysfunction and oxidative stress	Hebb‐Williams maze, Elevated Plus Maze (EPM), biochemical estimation of Na^+^/K^+^‐ATPase, TBARS, and AOPP	Chronic stress impaired cognitive function and reduced Na^+^/K^+^‐ATPase activity with altered oxidative stress markers. Enhanced learning and memory performance, restored Na^+^/K^+^‐ATPase activity, and normalized TBARS levels	Relevant to stress‐associated cognitive decline and oxidative neuronal damage implicated in neurodegenerative and psychiatric disorders	Rhizome powder	Not specified	Oral	21 days	Suggests neuroprotective and antioxidant potential against chronic stress‐induced cognitive impairment; further dose standardization and human safety studies are necessary	[18]
Mice	Scopolamine‐induced Alzheimer's type dementia (1 mg/kg, i.p.)	Morris Water Maze (MWM), Elevated Plus Maze (EPM), locomotor activity assessment, biochemical estimation of AChE, SOD, GSH, nitrite levels, histopathological analysis	Scopolamine caused cognitive impairment, oxidative stress, and increased AChE activity. Treatment with hydro‐alcoholic extract improved behavioral performance, restored antioxidant levels (SOD and GSH), reduced AChE activity, and provided neuroprotection in hippocampal tissue. Combination treatment with *Cordia dichotoma* showed enhanced protective effects	Mimics cholinergic dysfunction, oxidative stress, and neuronal degeneration associated with Alzheimer's disease and other cognitive disorders	Hydro‐alcoholic (1:1) extract of *A. calamus* alone and in combination with *Cordia dichotoma*	*A. calam*us: 600 mg/kg p.o.; *Cordia dichotoma*: 750 mg/kg p.o.	Oral (p.o.)	Not specified	Suggested anti‐amnesic, antioxidant, cholinergic, and neuroprotective effects of *A. calamus* for cognitive and neurodegenerative disorders; further clinical and toxicological validation is necessary before therapeutic application in humans	[22]

## 12 Regulatory Limits on Use of *A. calamus* and *β*‐asarone

Due to safety issues related with the use of *β*‐asarone, regulatory authorities such as U.S. Food and Drug Administration (US FDA) and The European Medicines Agency (EU) imposed limit on application of it. US FDA strictly banned the use of *β*‐asarone and calamus in food and related product due to possible carcinogenic effects. Similarly, EU, suggested the herbal medicine products containing *β*‐asarone should not exceed the limit 2 µg/kg body weight/day (≈115 µg/day). These regulatory prohibitions underline the importance of safety concern related with *β*‐asarone and signify the urgent necessary for its standardization and selection of chemotype for further application in pharmacology [104, 107]. In Table 4, details on chemotype, *β*‐asarone, genotoxicity concern and with regulatory measures were mentioned.

**TABLE 4:** Animal models used in study and risk of bias (qualitative) and behavioral assay consistency in *A. calamus*.

Risk of bias (qualitative)					Behavioral assay consistency			Reference
Randomization	Blinding	Sample size justified	Control used	Risk of bias	Test used	Validation status	Consistency across studies	
Not clearly reported	Not reported	Not justified	Noise stress and normal control groups	Moderate	Radial Eight‐arm Maze (RAM), antioxidant assays, AChE, Hsp70	Extensively validated behavioral and biochemical models for cognitive stress studies	Findings consistent with antioxidant and neuroprotective activity reported in other *A. calamus* studies	[79]
Not clearly reported	Not reported	Not justified	Yes	Moderate to high	conditioned avoidance response, spontaneous motor activity, hypothermia, anticonvulsant tests	Standard CNS pharmacological screening methods	CNS depressant and anticonvulsant results align with later neuropharmacological studies	[76]
Not clearly reported	Not reported	Not justified	standard drug Bilobil used	Moderate	open field test, tail suspension test, forced swim test, thiopental anesthesia	Standard validated psychotropic and behavioral assays	Behavioral findings moderately consistent with anxiolytic and CNS modulatory studies	[77]
Not clearly reported	Not reported	Not justified	LPS control	Moderate	Shuttle box test, Elevated Plus Maze (EPM)	Established neuroinflammation and memory assessment models	Consistent with anti‐inflammatory and antioxidant neuroprotection studies	[78]
Animals divided into groups, but randomization method not detailed	Not reported	sample size provided (n = 8/group), but no power calculation	vehicle, scopolamine, physostigmine controls	Moderate	Elevated plus‐maze, passive avoidance, shuttle‐box active avoidance, brain AChE assay	highly validated Alzheimer's disease‐related behavioral and cholinergic models	strongly consistent with anti‐amnesic and cholinergic enhancement studies of α‐asarone	[20]
Not clearly reported	Not reported	Not justified	Yes	Moderate	Hebb‐Williams maze, Elevated Plus Maze, oxidative stress assays	Widely accepted stress and memory models	Consistent with antioxidant‐mediated cognitive protection findings	[18]
Not clearly reported	Not reported	Not justified	Scopolamine control and treatment comparison groups	Moderate	Morris Water Maze (MWM), Elevated Plus Maze (EPM), locomotor activity, histopathology	Standard validated dementia and oxidative stress models	Consistent with anti‐amnesic, antioxidant, and neuroprotective evidence reported across studies	[22]

Additional efforts are necessary to thoroughly evaluate the toxicology of α‐asarone, β‐asarone, and γ‐asarone (Table 5). The toxicokinetic data available today is still lacking, especially when it comes to species‐specific variations. To comprehensively assess their negative effects and clarify the underlying mechanisms of toxicity, it is therefore essential to generate high‐quality toxicological data in accordance with Organization for Economic Cooperation and Development test requirements.

**TABLE 5:** Ploidy level, β‐asarone, and chemotype of *A. calamus* with regulatory measures.

Ploidy level	β‐asarone content	Geographic distribution	Toxicity risk	Genotoxicity concern	Regulatory acceptance	Suitability for medicinal use	Reference
Diploid (2n)	Absent/trace	North America	Low	Minimal	Preferred	Safer option	[50, 51, 52, 54, 60, 108, 109, 110]
Triploid (3n)	Moderate to high	Europe	Moderate	Present	Restricted	Limited use	[50, 54, 58]
Tetraploid (4n)	High	Asia (India, China)	High	Significant	Highly restricted	Not recommended	[50, 58]

## 13 Conclusion and Future Prospective

The current review focuses on pharmacological potential of *A. calamus* specifically on cognitive property. Besides these, the present review also highlighted the ethonobotanical aspects of the plant with scientific evidence. The key chemical present in the essential oil of the *A. calamus* is *β*‐asarone followed by camphene, (E) *β* ocimene, camphor, calarene, α cadinol, and selinene. This review highlights ethnobotanical aspects along with *in silico* validated reports till date on various pharmacological applications. The most promising therapeutic target of *A. calamus* is cognitive and neurological related applications, since the species is used from earlier times to treat above said disorders. Previously, a number of studies were reported on in vitro, in vivo, and *in silico* aspects on neurological problems. However, rigorous and detail *in vivo* studies are required for the validation of reported *in vitro* studies. Further, most urgently extensive studies on positive and negative aspects of marker compound such as *β*‐asarone, *α*‐asarone is required for clinical trials and to validate its long practiced ethnobotanical applications on cognitive and neurological disorders. Moreover, this review will highlight the significance of *A. calamus* in cognitive enhancement and related research near future.

## Author Contribution Statement

**R. T**.: Writing – original draft, investigation, methodology, formal analysis. **A. G**.: Methodology, investigation. **T. B**.: Conceptualization, supervision, writing – review and editing. **M. L**.: project administration, funding acquisition, writing – review and editing.

## Funding

The authors are thankful to the CSIR ‐ New Delhi for funding in the form of project number HCP‐007.

## Conflicts of Interest

The authors declare no conflicts of interest.

## Data Availability

In the present review no data were produced during the study.
